# *N*-(1,5-Dimethyl-3-oxo-2-phenyl-2,3-dihydro-1*H*-pyrazol-4-yl)acetamide–naphthalene-2,3-diol (1/1)

**DOI:** 10.1107/S1600536810024438

**Published:** 2010-06-26

**Authors:** Abdullah M. Asiri, Salman A. Khan, Kong Wai Tan, Seik Weng Ng

**Affiliations:** a Chemistry Department, Faculty of Science, King Abdul Aziz University, PO Box 80203, Jeddah 21589, Saudi Arabia; b Department of Chemistry, University of Malaya, 50603 Kuala Lumpur, Malaysia

## Abstract

In the reaction of naphthalene-2,3-diol and 4-amino­anti­pyrine in the presence of acetic acid, the amine function is acetyl­ated and the resulting acetamide co-crystallizes with the diol in the title compound, C_13_H_15_N_3_O_2_·C_10_H_8_O_2_, with 1:1 molar stoichiometry. The two components are linked by two O–H⋯O=C hydrogen bonds. One of the hy­droxy groups inter­acts with the pyrazolone carbonyl O atom and the other hy­droxy group inter­acts with the amide O atom of another component, generating a chain motif. Adjacent chains are linked into a layer motif *via* N—H⋯O inter­actions involving only the heterocyclic acetamide component.

## Related literature

For the crystal structure of 4-acetamido-2,3-dimethyl-1-phenyl-5-pyrazol-3-one, see: Kuznetsov *et al.* (1999 ▶). For co-crystals of naphthalene-2,3-diol, see: Fritchie & Johnston (1975 ▶); Herbert & Truter (1980 ▶); Kuo *et al.* (1974 ▶); Nakamatsu *et al.* (2003 ▶); Wang *et al.* (2008 ▶); Wells *et al.* (1974 ▶).
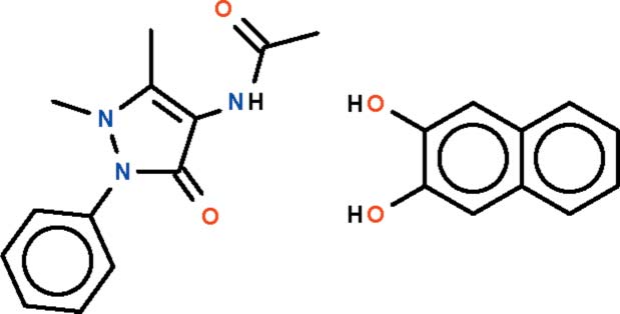


## Experimental

### 

#### Crystal data

C_13_H_15_N_3_O_2_·C_10_H_8_O_2_
*M* *_r_* = 405.44
Monoclinic, 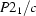

*a* = 12.426 (1) Å
*b* = 14.304 (2) Å
*c* = 12.959 (1) Å
β = 117.845 (1)°
*V* = 2036.7 (4) Å^3^
*Z* = 4
Mo *K*α radiation
μ = 0.09 mm^−1^
*T* = 100 K
0.25 × 0.25 × 0.10 mm

#### Data collection

Bruker SMART APEX diffractometer
19263 measured reflections
4683 independent reflections
3189 reflections with *I* > 2σ(*I*)
*R*_int_ = 0.061

#### Refinement

*R*[*F* ^2^ > 2σ(*F* ^2^)] = 0.052
*wR*(*F* ^2^) = 0.131
*S* = 1.02
4683 reflections
286 parameters
27 restraints
H atoms treated by a mixture of independent and constrained refinement
Δρ_max_ = 0.31 e Å^−3^
Δρ_min_ = −0.36 e Å^−3^

### 

Data collection: *APEX2* (Bruker, 2009 ▶); cell refinement: *SAINT* (Bruker, 2009 ▶); data reduction: *SAINT*; program(s) used to solve structure: *SHELXS97* (Sheldrick, 2008 ▶); program(s) used to refine structure: *SHELXL97* (Sheldrick, 2008 ▶); molecular graphics: *X-SEED* (Barbour, 2001 ▶); software used to prepare material for publication: *publCIF* (Westrip, 2010 ▶).

## Supplementary Material

Crystal structure: contains datablocks global, I. DOI: 10.1107/S1600536810024438/im2213sup1.cif

Structure factors: contains datablocks I. DOI: 10.1107/S1600536810024438/im2213Isup2.hkl

Additional supplementary materials:  crystallographic information; 3D view; checkCIF report

## Figures and Tables

**Table 1:** Hydrogen-bond geometry (Å, °)

*D*—H⋯*A*	*D*—H	H⋯*A*	*D*⋯*A*	*D*—H⋯*A*
N1—H1⋯O2^i^	0.87 (1)	2.07 (1)	2.924 (2)	169 (2)
O3—H3⋯O2	0.85 (3)	1.81 (3)	2.639 (2)	163 (3)
O4—H4⋯O1^ii^	0.85 (3)	1.81 (3)	2.646 (2)	168 (3)

Symmetry codes: (i) 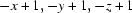; (ii) 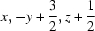.

**Fig. 1.**
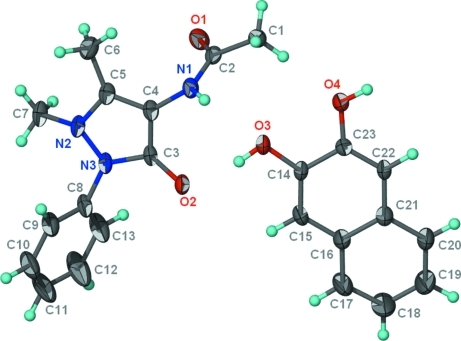
Thermal ellipsoid plot (Barbour, 2001) of the (C13H15N3O2)(C10H8O2) co-crystal at the 70% probability level; hydrogen atoms are drawn as spheres of arbitrary radius.

## supplementary crystallographic information

### Comment

Naphthalene-2,3-diol forms co-crystals with a number of neutral organic compounds (Fritchie & Johnston, 1975; Herbert & Truter, 1980; Kuo *et al.*, 1974; Nakamatsu *et al.*, 2003; Wang *et al.*, 2008; Wells *et al.*, 1974). The attempt to co-crystallize it with the drug 4-aminoantipyrine was sussessful when the reaction was carried out in the presence of acetic acid, but the acetic acid converted the amino group to an acetamido group instead. In the co-crystal (Scheme I, Fig. 1), the acetamide and the diol are linked by two *O*–H···O═C hydrogen bonds. One of the hydroxy groups interacts with the pyrazolyl carbonyl O-atom and the other hydroxy group interacts with the amido O-atom of another component to generate a chain motif. Adjacent chains are linked into a layer motif *via* N–H···O interactions that involves the acetamide component only.

### Experimental

Naphthalene-2,3-diol (0.35 g, 2.2 mol) and 4-aminoantipyrine (0.45 g, 2.2 mmol) were heated in methanol (15 ml) for 5 h; three drops of acetic acid were added. Crystals separated from the cool solution when it was set aside for a day.

### Refinement

Carbon-bound H-atoms were placed in calculated positions [C–H 0.95 to 0.98 Å, *U*(H) = 1.2 to 1.5*U*_eq_(C)] and were included in the refinement in the riding model approximation. The hydroxy and amino H-atoms were located in a difference Fourier map, and were refined with distance restraints of O–H 0.84±0.01Å and N–H 0.86±0.01 Å. Their temperature factors were freely refined.

The anisotropic temperature factors of C17 to C20 atoms of the naphthalene fused-ring were restrained to be nearly isotropic and with the restraints, the ellipsoids were somewhat elongated.

### Crystal data

**Table:**

C_13_H_15_N_3_O_2_·C_10_H_8_O_2_	*F*(000) = 856
*M**_r_* = 405.44	*D*_x_ = 1.322 Mg m^−^^3^
Monoclinic, *P*2_1_/*c*	Mo *K*α radiation, λ = 0.71073 Å
Hall symbol: -P 2ybc	Cell parameters from 2505 reflections
*a* = 12.426 (1) Å	θ = 2.3–27.9°
*b* = 14.304 (2) Å	µ = 0.09 mm^−^^1^
*c* = 12.959 (1) Å	*T* = 100 K
β = 117.845 (1)°	Prism, colorless
*V* = 2036.7 (4) Å^3^	0.25 × 0.25 × 0.10 mm
*Z* = 4	

### Data collection

**Table:**

Bruker SMART APEX diffractometer	3189 reflections with *I* > 2σ(*I*)
Radiation source: fine-focus sealed tube	*R*_int_ = 0.061
graphite	θ_max_ = 27.5°, θ_min_ = 1.9°
ω scans	*h* = −16→16
19263 measured reflections	*k* = −18→18
4683 independent reflections	*l* = −16→16

### Refinement

**Table:**

Refinement on *F*^2^	Primary atom site location: structure-invariant direct methods
Least-squares matrix: full	Secondary atom site location: difference Fourier map
*R*[*F*^2^ > 2σ(*F*^2^)] = 0.052	Hydrogen site location: inferred from neighbouring sites
*wR*(*F*^2^) = 0.131	H atoms treated by a mixture of independent and constrained refinement
*S* = 1.02	*w* = 1/[σ^2^(*F*_o_^2^) + (0.0544*P*)^2^ + 0.6929*P*] where *P* = (*F*_o_^2^ + 2*F*_c_^2^)/3
4683 reflections	(Δ/σ)_max_ = 0.001
286 parameters	Δρ_max_ = 0.31 e Å^−^^3^
27 restraints	Δρ_min_ = −0.35 e Å^−^^3^

### Fractional atomic coordinates and isotropic or equivalent isotropic displacement parameters (Å^2^)

**Table:**

	*x*	*y*	*z*	*U*_iso_*/*U*_eq_	
O1	0.33173 (12)	0.73209 (10)	0.32885 (12)	0.0290 (3)	
O2	0.65387 (11)	0.56345 (9)	0.48381 (11)	0.0232 (3)	
O3	0.56600 (12)	0.65279 (10)	0.60626 (11)	0.0253 (3)	
O4	0.44923 (12)	0.70985 (10)	0.71521 (12)	0.0279 (3)	
N1	0.37471 (14)	0.57790 (11)	0.36783 (14)	0.0224 (4)	
N2	0.51486 (14)	0.55398 (11)	0.18066 (13)	0.0233 (4)	
N3	0.61709 (14)	0.55929 (11)	0.29124 (13)	0.0219 (4)	
C1	0.25557 (17)	0.65611 (14)	0.44515 (17)	0.0255 (4)	
H1A	0.1879	0.7007	0.4123	0.038*	
H1B	0.3105	0.6728	0.5264	0.038*	
H1C	0.2237	0.5929	0.4422	0.038*	
C2	0.32386 (16)	0.65897 (14)	0.37571 (16)	0.0219 (4)	
C3	0.58105 (17)	0.56524 (12)	0.37598 (16)	0.0199 (4)	
C4	0.45083 (17)	0.57041 (13)	0.31423 (16)	0.0218 (4)	
C5	0.41487 (17)	0.56533 (13)	0.19785 (16)	0.0240 (4)	
C6	0.29135 (19)	0.57192 (16)	0.09672 (18)	0.0326 (5)	
H6A	0.2302	0.5591	0.1226	0.049*	
H6B	0.2835	0.5261	0.0374	0.049*	
H6C	0.2788	0.6350	0.0634	0.049*	
C7	0.52538 (19)	0.60264 (14)	0.08616 (17)	0.0284 (5)	
H7A	0.4540	0.5885	0.0116	0.043*	
H7B	0.5992	0.5817	0.0836	0.043*	
H7C	0.5300	0.6702	0.1002	0.043*	
C8	0.73390 (18)	0.53349 (14)	0.30516 (17)	0.0266 (4)	
C9	0.7468 (2)	0.45251 (15)	0.25275 (18)	0.0326 (5)	
H9	0.6785	0.4135	0.2091	0.039*	
C10	0.8600 (2)	0.4293 (2)	0.2648 (2)	0.0501 (7)	
H10	0.8696	0.3745	0.2285	0.060*	
C11	0.9594 (2)	0.4857 (2)	0.3296 (3)	0.0681 (10)	
H11	1.0371	0.4698	0.3375	0.082*	
C12	0.9456 (2)	0.5654 (2)	0.3830 (3)	0.0676 (10)	
H12	1.0144	0.6035	0.4284	0.081*	
C13	0.8323 (2)	0.59026 (18)	0.3707 (2)	0.0428 (6)	
H13	0.8226	0.6452	0.4067	0.051*	
C14	0.63471 (17)	0.66192 (13)	0.72372 (15)	0.0202 (4)	
C15	0.75639 (18)	0.64515 (14)	0.78429 (17)	0.0266 (4)	
H15	0.7987	0.6232	0.7440	0.032*	
C16	0.82114 (19)	0.65991 (16)	0.90656 (18)	0.0339 (5)	
C17	0.9490 (2)	0.6515 (2)	0.9714 (2)	0.0634 (9)	
H17	0.9945	0.6316	0.9335	0.076*	
C18	1.0082 (3)	0.6715 (3)	1.0883 (2)	0.0872 (13)	
H18	1.0944	0.6674	1.1299	0.105*	
C19	0.9427 (2)	0.6980 (3)	1.1467 (2)	0.0719 (10)	
H19	0.9845	0.7104	1.2281	0.086*	
C20	0.8197 (2)	0.70618 (18)	1.08762 (19)	0.0406 (6)	
H20	0.7761	0.7235	1.1285	0.049*	
C21	0.75571 (18)	0.68925 (14)	0.96659 (17)	0.0274 (4)	
C22	0.62919 (17)	0.70463 (13)	0.90182 (16)	0.0211 (4)	
H22	0.5848	0.7235	0.9413	0.025*	
C23	0.56923 (16)	0.69285 (12)	0.78342 (16)	0.0198 (4)	
H1	0.373 (2)	0.5318 (11)	0.4107 (17)	0.036 (6)*	
H3	0.608 (2)	0.6281 (17)	0.577 (2)	0.054 (8)*	
H4	0.419 (2)	0.7345 (19)	0.755 (2)	0.067 (9)*	

### Atomic displacement parameters (Å^2^)

**Table:**

	*U*^11^	*U*^22^	*U*^33^	*U*^12^	*U*^13^	*U*^23^
O1	0.0304 (8)	0.0313 (8)	0.0338 (8)	0.0020 (6)	0.0222 (7)	0.0056 (6)
O2	0.0239 (7)	0.0308 (7)	0.0178 (7)	−0.0018 (6)	0.0121 (6)	−0.0039 (5)
O3	0.0269 (7)	0.0340 (8)	0.0172 (7)	0.0026 (6)	0.0121 (6)	−0.0035 (6)
O4	0.0215 (7)	0.0423 (9)	0.0207 (7)	0.0040 (6)	0.0106 (6)	−0.0036 (6)
N1	0.0230 (8)	0.0287 (9)	0.0210 (9)	−0.0039 (7)	0.0147 (7)	0.0004 (7)
N2	0.0266 (9)	0.0303 (9)	0.0165 (8)	−0.0067 (7)	0.0131 (7)	−0.0030 (7)
N3	0.0238 (8)	0.0281 (9)	0.0175 (8)	−0.0064 (7)	0.0127 (7)	−0.0067 (7)
C1	0.0186 (9)	0.0374 (11)	0.0224 (10)	0.0005 (8)	0.0111 (8)	0.0010 (8)
C2	0.0169 (9)	0.0310 (10)	0.0170 (9)	−0.0037 (8)	0.0073 (8)	−0.0025 (8)
C3	0.0263 (10)	0.0194 (9)	0.0196 (10)	−0.0040 (7)	0.0155 (8)	−0.0036 (7)
C4	0.0246 (10)	0.0240 (10)	0.0214 (10)	−0.0042 (8)	0.0147 (8)	−0.0025 (8)
C5	0.0273 (10)	0.0275 (10)	0.0203 (10)	−0.0063 (8)	0.0137 (8)	−0.0024 (8)
C6	0.0285 (11)	0.0463 (13)	0.0221 (11)	−0.0047 (10)	0.0110 (9)	−0.0027 (9)
C7	0.0387 (12)	0.0323 (11)	0.0211 (10)	−0.0084 (9)	0.0198 (10)	−0.0035 (8)
C8	0.0275 (10)	0.0352 (11)	0.0251 (11)	−0.0061 (8)	0.0191 (9)	−0.0079 (8)
C9	0.0346 (12)	0.0400 (12)	0.0291 (12)	−0.0036 (10)	0.0198 (10)	−0.0093 (9)
C10	0.0452 (15)	0.0678 (18)	0.0458 (15)	0.0041 (13)	0.0284 (13)	−0.0223 (13)
C11	0.0308 (14)	0.117 (3)	0.0654 (19)	−0.0044 (15)	0.0300 (14)	−0.0417 (18)
C12	0.0341 (14)	0.110 (3)	0.071 (2)	−0.0277 (15)	0.0349 (14)	−0.0542 (19)
C13	0.0349 (13)	0.0565 (15)	0.0477 (15)	−0.0169 (11)	0.0282 (12)	−0.0290 (12)
C14	0.0257 (10)	0.0207 (9)	0.0159 (9)	−0.0005 (8)	0.0111 (8)	−0.0022 (7)
C15	0.0262 (10)	0.0348 (11)	0.0247 (11)	0.0040 (8)	0.0169 (9)	−0.0030 (8)
C16	0.0252 (11)	0.0538 (14)	0.0230 (11)	0.0065 (10)	0.0115 (9)	−0.0049 (10)
C17	0.0274 (13)	0.125 (3)	0.0358 (14)	0.0178 (14)	0.0129 (11)	−0.0205 (15)
C18	0.0297 (15)	0.180 (4)	0.0390 (16)	0.0292 (19)	0.0051 (13)	−0.025 (2)
C19	0.0341 (14)	0.142 (3)	0.0275 (14)	0.0191 (16)	0.0041 (11)	−0.0231 (16)
C20	0.0315 (12)	0.0672 (17)	0.0221 (11)	0.0076 (11)	0.0117 (10)	−0.0076 (11)
C21	0.0251 (10)	0.0361 (11)	0.0215 (10)	0.0012 (9)	0.0115 (9)	−0.0030 (8)
C22	0.0259 (10)	0.0235 (9)	0.0186 (9)	−0.0005 (8)	0.0145 (8)	−0.0003 (7)
C23	0.0201 (9)	0.0205 (9)	0.0197 (10)	0.0000 (7)	0.0101 (8)	−0.0004 (7)

### Geometric parameters (Å, °)

**Table:**

O1—C2	1.236 (2)	C8—C9	1.389 (3)
O2—C3	1.261 (2)	C9—C10	1.381 (3)
O3—C14	1.360 (2)	C9—H9	0.9500
O3—H3	0.85 (3)	C10—C11	1.383 (4)
O4—C23	1.354 (2)	C10—H10	0.9500
O4—H4	0.85 (3)	C11—C12	1.385 (4)
N1—C2	1.347 (2)	C11—H11	0.9500
N1—C4	1.415 (2)	C12—C13	1.388 (3)
N1—H1	0.868 (10)	C12—H12	0.9500
N2—C5	1.371 (2)	C13—H13	0.9500
N2—N3	1.404 (2)	C14—C15	1.361 (3)
N2—C7	1.466 (2)	C14—C23	1.430 (2)
N3—C3	1.369 (2)	C15—C16	1.418 (3)
N3—C8	1.425 (2)	C15—H15	0.9500
C1—C2	1.498 (2)	C16—C17	1.413 (3)
C1—H1A	0.9800	C16—C21	1.426 (3)
C1—H1B	0.9800	C17—C18	1.369 (4)
C1—H1C	0.9800	C17—H17	0.9500
C3—C4	1.433 (3)	C18—C19	1.398 (4)
C4—C5	1.360 (3)	C18—H18	0.9500
C5—C6	1.484 (3)	C19—C20	1.358 (3)
C6—H6A	0.9800	C19—H19	0.9500
C6—H6B	0.9800	C20—C21	1.409 (3)
C6—H6C	0.9800	C20—H20	0.9500
C7—H7A	0.9800	C21—C22	1.411 (3)
C7—H7B	0.9800	C22—C23	1.367 (3)
C7—H7C	0.9800	C22—H22	0.9500
C8—C13	1.380 (3)		
			
C14—O3—H3	109.9 (18)	C10—C9—H9	120.4
C23—O4—H4	110.2 (19)	C8—C9—H9	120.4
C2—N1—C4	123.16 (16)	C9—C10—C11	120.1 (2)
C2—N1—H1	117.1 (15)	C9—C10—H10	119.9
C4—N1—H1	118.4 (15)	C11—C10—H10	119.9
C5—N2—N3	106.52 (14)	C10—C11—C12	120.0 (2)
C5—N2—C7	121.38 (17)	C10—C11—H11	120.0
N3—N2—C7	115.82 (15)	C12—C11—H11	120.0
C3—N3—N2	110.07 (14)	C11—C12—C13	120.6 (2)
C3—N3—C8	127.60 (16)	C11—C12—H12	119.7
N2—N3—C8	119.84 (14)	C13—C12—H12	119.7
C2—C1—H1A	109.5	C8—C13—C12	118.7 (2)
C2—C1—H1B	109.5	C8—C13—H13	120.7
H1A—C1—H1B	109.5	C12—C13—H13	120.7
C2—C1—H1C	109.5	O3—C14—C15	125.21 (16)
H1A—C1—H1C	109.5	O3—C14—C23	114.64 (16)
H1B—C1—H1C	109.5	C15—C14—C23	120.15 (17)
O1—C2—N1	122.93 (17)	C14—C15—C16	121.07 (17)
O1—C2—C1	121.01 (17)	C14—C15—H15	119.5
N1—C2—C1	116.05 (17)	C16—C15—H15	119.5
O2—C3—N3	123.60 (17)	C17—C16—C15	123.06 (19)
O2—C3—C4	131.17 (16)	C17—C16—C21	117.99 (19)
N3—C3—C4	105.21 (15)	C15—C16—C21	118.87 (18)
C5—C4—N1	126.85 (17)	C18—C17—C16	121.0 (2)
C5—C4—C3	108.47 (16)	C18—C17—H17	119.5
N1—C4—C3	124.68 (16)	C16—C17—H17	119.5
C4—C5—N2	109.47 (17)	C17—C18—C19	120.5 (2)
C4—C5—C6	130.11 (18)	C17—C18—H18	119.8
N2—C5—C6	120.40 (17)	C19—C18—H18	119.8
C5—C6—H6A	109.5	C20—C19—C18	120.3 (2)
C5—C6—H6B	109.5	C20—C19—H19	119.8
H6A—C6—H6B	109.5	C18—C19—H19	119.8
C5—C6—H6C	109.5	C19—C20—C21	120.9 (2)
H6A—C6—H6C	109.5	C19—C20—H20	119.5
H6B—C6—H6C	109.5	C21—C20—H20	119.5
N2—C7—H7A	109.5	C20—C21—C22	121.83 (18)
N2—C7—H7B	109.5	C20—C21—C16	119.28 (19)
H7A—C7—H7B	109.5	C22—C21—C16	118.82 (18)
N2—C7—H7C	109.5	C23—C22—C21	121.33 (17)
H7A—C7—H7C	109.5	C23—C22—H22	119.3
H7B—C7—H7C	109.5	C21—C22—H22	119.3
C13—C8—C9	121.35 (19)	O4—C23—C22	124.57 (16)
C13—C8—N3	118.83 (18)	O4—C23—C14	115.71 (16)
C9—C8—N3	119.81 (18)	C22—C23—C14	119.71 (17)
C10—C9—C8	119.3 (2)		
			
C5—N2—N3—C3	5.4 (2)	C8—C9—C10—C11	0.8 (4)
C7—N2—N3—C3	143.68 (16)	C9—C10—C11—C12	0.3 (5)
C5—N2—N3—C8	168.77 (16)	C10—C11—C12—C13	−1.0 (5)
C7—N2—N3—C8	−52.9 (2)	C9—C8—C13—C12	0.5 (4)
C4—N1—C2—O1	5.8 (3)	N3—C8—C13—C12	−179.3 (2)
C4—N1—C2—C1	−174.38 (16)	C11—C12—C13—C8	0.6 (5)
N2—N3—C3—O2	174.23 (16)	O3—C14—C15—C16	177.70 (19)
C8—N3—C3—O2	12.4 (3)	C23—C14—C15—C16	−1.5 (3)
N2—N3—C3—C4	−4.09 (19)	C14—C15—C16—C17	−174.2 (2)
C8—N3—C3—C4	−165.87 (17)	C14—C15—C16—C21	2.5 (3)
C2—N1—C4—C5	−79.6 (3)	C15—C16—C17—C18	176.3 (3)
C2—N1—C4—C3	101.6 (2)	C21—C16—C17—C18	−0.4 (5)
O2—C3—C4—C5	−176.82 (19)	C16—C17—C18—C19	2.0 (6)
N3—C3—C4—C5	1.3 (2)	C17—C18—C19—C20	−1.5 (6)
O2—C3—C4—N1	2.2 (3)	C18—C19—C20—C21	−0.7 (5)
N3—C3—C4—N1	−179.66 (17)	C19—C20—C21—C22	−174.8 (3)
N1—C4—C5—N2	−176.99 (17)	C19—C20—C21—C16	2.3 (4)
C3—C4—C5—N2	2.0 (2)	C17—C16—C21—C20	−1.7 (3)
N1—C4—C5—C6	4.2 (3)	C15—C16—C21—C20	−178.5 (2)
C3—C4—C5—C6	−176.78 (19)	C17—C16—C21—C22	175.4 (2)
N3—N2—C5—C4	−4.4 (2)	C15—C16—C21—C22	−1.4 (3)
C7—N2—C5—C4	−139.91 (18)	C20—C21—C22—C23	176.4 (2)
N3—N2—C5—C6	174.48 (17)	C16—C21—C22—C23	−0.7 (3)
C7—N2—C5—C6	39.0 (3)	C21—C22—C23—O4	−177.26 (18)
C3—N3—C8—C13	−64.6 (3)	C21—C22—C23—C14	1.7 (3)
N2—N3—C8—C13	135.2 (2)	O3—C14—C23—O4	−0.8 (2)
C3—N3—C8—C9	115.5 (2)	C15—C14—C23—O4	178.42 (17)
N2—N3—C8—C9	−44.7 (3)	O3—C14—C23—C22	−179.90 (16)
C13—C8—C9—C10	−1.2 (3)	C15—C14—C23—C22	−0.6 (3)
N3—C8—C9—C10	178.6 (2)		

### Hydrogen-bond geometry (Å, °)

**Table:**

*D*—H···*A*	*D*—H	H···*A*	*D*···*A*	*D*—H···*A*
N1—H1···O2^i^	0.87 (1)	2.07 (1)	2.924 (2)	169 (2)
O3—H3···O2	0.85 (3)	1.81 (3)	2.639 (2)	163 (3)
O4—H4···O1^ii^	0.85 (3)	1.81 (3)	2.646 (2)	168 (3)

Symmetry codes: (i) −*x*+1, −*y*+1, −*z*+1; (ii) *x*, −*y*+3/2, *z*+1/2.
